# Synthesis and magnetic properties of Zr doped ZnO Nanoparticles

**DOI:** 10.1186/1556-276X-6-587

**Published:** 2011-11-10

**Authors:** Jing Zhang, Daqiang Gao, Guijin Yang, Jinlin Zhang, Zhenhua Shi, Zhaohui Zhang, Zhonghua Zhu, Desheng Xue

**Affiliations:** 1 Key Laboratory for Magnetism and Magnetic Materials of MOE, Lanzhou University, Lanzhou 730000, PR China

**Keywords:** Zn_1-x_Zr_x_O nanoparticles, Room temperature ferromagnetism, Oxygen vacancies

## Abstract

Zr doped ZnO nanoparticles are prepared by the sol-gel method with post-annealing. X-ray diffraction results show that all samples are the typical hexagonal wurtzite structure without any other new phase, as well as the Zr atoms have successfully entered into the ZnO lattices instead of forming other lattices. Magnetic measurements indicate that all the doping samples show room temperature ferromagnetism and the pure ZnO is paramagneism. The results of Raman and X-ray photoelectron spectroscopy indicate that there are a lot of oxygen vacancies in the samples by doping element of Zr. It is considered that the observed ferromagnetism is related to the doping induced oxygen vacancies.

## Introduction

Diluted magnetic semiconductors (DMSs) have attracted intense interest due to their potential applications in spintronic devices [1-3]. DMSs are usually produced by doping semiconductors with transition metals (TMs). Through theoretically predicting, GaN and ZnO as typical n-type semiconductors would be ideal candidates for room-temperature (RT) DMSs [4]. The room temperature ferromagnetism (RTFM) in TM-doped GaN has been reported in experiment and theroy, such as, Mn [5,6], Gd [7], and Cr [8,9]. Compared with GaN, ZnO has a lot of outstanding superiorities, as is known to all, which has a wide band-gap (3.37 eV at RT) and a high excitation binding energy (60 meV at RT), so ZnO has been got more and more attention. Otherwise, since Dietl *et al*. predicted that Mn-doped ZnO can show the clear RTFM and also has a higher Curie temperature (*T_C_*) than RT [10], which triggered worldwide interest in research of the doping ZnO materials. At first, RTFM has been demonstrated for various kinds of TM-doped ZnO, for example, Mn [11], Co [12], Ni [13], and Fe [14]. However, the origin of their magnetism remains controversy, because it is not yet clear whether the observed RTFM is truly intrinsic or related to secondary phases such as clusters [13]. To avoid the impact from ferromagnetic (FM) elements, in recent years, RTFM in ZnO doping with other non-ferromagnetic elements has been discovered in experiment and theory, for instance, Cu [15,16], V [17], Cr [18,19], Li [20,21], C [22], Er [23]-doped ZnO. However, until now there is no consensus on the origin of FM in doping ZnO, so we researched the origin of RTFM in the doping ZnO materials, it was hoped that we could get a better explanation about this intractable issue.

Paul *et al*. prepared the Zr doped ZnO films using a sol-gel technique with post-annealing successfully and found the films of extremely great properties, such as in the structural, optical, and electrical aspects, otherwise, at higher Zr concentrations, increasing dopant atom forms some kinds of defects [24]. Defects may cause FM to appear reported before [15,23], so in this paper, we prepared Zr doped ZnO nanoparticles (NPs) by the same method and studied the structure and their magnetic property with the different Zr doping contents.

## Experiment

Zn_1-x_Zr_x_O NPs were prepared by the sol-gel method with post-annealing. All the chemical reagents used as starting materials are analytic grade reagents and purchased without any further treatment. Firstly, 0.1 *M *Zn(NO_3_)_2_·6H_2_O and y *M *(y = 0.0005, 0.001, 0.0015, and 0.002) Zr(NO_3_)_4_·5H_2_O were dissolved into the ethylene glycol monomethylether (C_3_H_8_O_2_). Then, the dissolved solution was stirred for 4 h at 80°C and dried at 80°C in the oven to form the precursor. Finally, the precursor was annealed at 500°C for 1.5 h in the air and the series of Zn_1-x_Zr_x_O NPs were obtained. At the same time, Zr contents of Zn_1-x_Zr_x_O samples are consistent with the mole percentage (x = 0.005, 0.01, 0.015, and 0.02).

The morphologies of samples were characterized by scanning electron microscope (SEM, Hitachi S-4800, Hitachi High Technologies America, Inc., Schaumburg, IL, USA) and transmission electron microscope (TEM, JEM-2010, JEOL Ltd., Tokyo, Japan). Selected area electron diffraction (SAED) and x-ray diffraction (XRD, X' Pert PRO PHILIPS with Cu Kα radiation, PANalytical, Shanghai, People's Republic of China) were employed to study the structure of the samples. The vibration properties were characterized by the Raman scattering spectra measurement, which was performed on a Jobin-Yvon LabRam HR80 spectrometer (Horiba Jobin Yvon Inc., Edison, NJ, USA) with a 325 nm line of Torus 50 mW diode-pumped solid-state laser under backscattering geometry. X-ray photoelectron spectroscopy (XPS, VG ESCALAB 210, VG Scientific Ltd., East Grinstead, UK) was utilized to determine the bonding characteristics and the composition of the particles. The measurements of magnetic properties were made using vibrating sample magnetometer (VSM, Lakeshore 7304, Lakeshore Cryotronics, Inc., Westerville, OH, USA) and Quantum Design MPMS magnetometer based on superconducting quantum interference device (SQUID).

## Results and discussion

**Figure 1:**
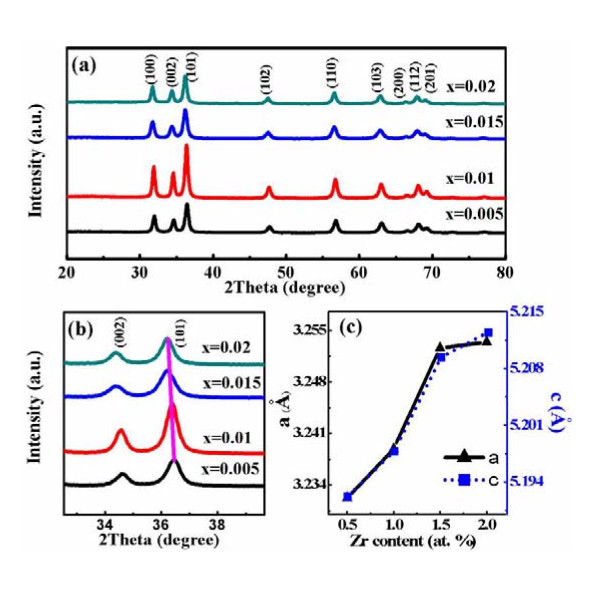
**XRD patterns represented by lines of different colors**. (**a**) XRD patterns of Zn_1-x_Zr_x_O samples; (**b**) XRD patterns of Zn_1-x_Zr_x_O samples in detail; (**c**) the variation of the lattice parameter *a *and *c *dependent on the Zr content in samples (x = 0.005, 0.01, 0.015, 0.02).

The XRD patterns of Zn_1-x_Zr_x_O samples (x = 0.005, 0.01, 0.015, 0.02) are shown in Figure 1(a). The results indicate that all the samples are the typical hexagonal wurtzite structure (JCPDS card no.36-1451). No phase of Zr or its oxide is observed. Figure 1(b) shows an observably slight shift towards the smaller angle with enhancing of the Zr doping content x. And the lattice parameter *a *and *c *increase monotonously with the content × increasing (shown in Figure 1(c)) based on the results of Figure 1(a). This reason may be that the ionic radius of Zr^4+ ^(0.84 Å) is larger than that of Zn^2+ ^(0.74 Å) [25,26], the more Zn^2+ ^were substituted by Zr^4+^, the greater lattice distortion of ZnO would be generated, the more lattice expansion would become. These results indicate that the Zr atoms have successfully entered into the ZnO lattices instead of forming other lattices.

**Figure 2:**
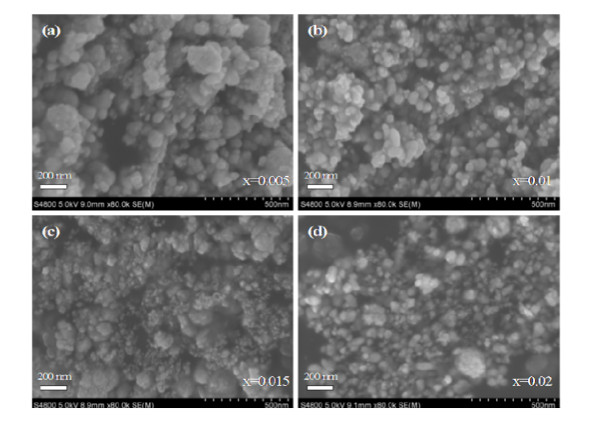
**SEM images of ZnO NPs with different Zr contents**.

**Figure 3:**
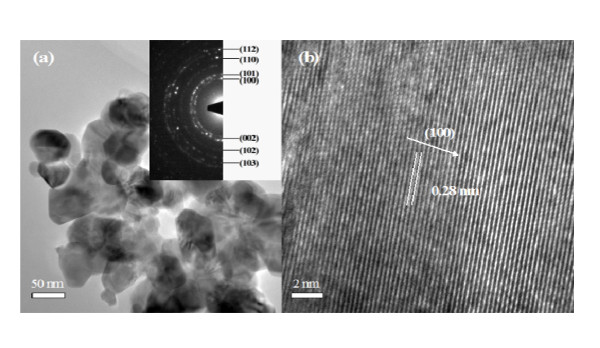
**TEM and HRTEM images of Zn_0.995_Zr_0.005_O Nps**. (**a**) The representative TEM image of Zn_0.995_Zr_0.005_O and the inset is the SAED pattern. (**b**) The HRTEM image of Zn_0.995_Zr_0.005_O.

Figure 2 shows the SEM images of Zn_1-x_Zr_x_O samples (x = 0.005, 0.01, 0.015, 0.02). It is clearly seen that all the Zn_1-x_Zr_x_O NPs are partly accumulated together with different sizes, while many little NPs with the about 60 nm diameter make up a comparatively bigger NP. Further, the size and shape of the NPs does not change a lot as the content × of Zr doping enhances. The particle morphologies for the samples were also obtained by the TEM images, Figure 3(a) shows the representative TEM image of Zn_0.995_Zr_0.005_O NPs which also confirms that NPs are accumulated together and the diameter of the NPs is about 60 nm. The homologous SAED pattern in the inset of Figure 3(a) shows discontinuous diffraction rings instead of shiny spots, which are attributed to the hexagonal wurtzite structured ZnO crystal and indicate that NPs are polycrystalline. It can be clearly seen from the high-resolution electron microscopy (HRTEM) image of Zn_0.995_Zr_0.005_O in Figure 3(b) that NPs are well crystallized and the interplanar spacing as calculated from the HRTEM image is 0.28 nm, corresponding to the lattice constant of the standard hexagonal wurtzite structured ZnO in (100) plane.

**Figure 4:**
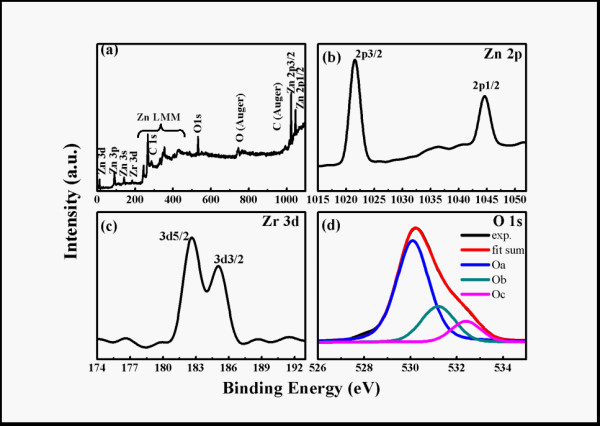
**XPS spectra represented by lines of different colors**. (a) XPS survey spectrum, high resolution scan of (b) Zn 2*p*, (c) Zr 3*d*, and (d) O 1*s *of Zn_0.995_Zr_0.005_O Nps.

The chemical states of the compositional elements in Zn_1-x_Zr_x_O NPs were revealed by the XPS and the representative spectra of Zn_0.995_Zr_0.005_O are shown in Figure 4. In Figure 4(a), the survey spectrum, the indexed peaks are only correspond to elements Zn, O, Zr, and C, where the binding energies are calibrated by taking carbon C 1*s *peak (284.6 eV). The peak located at 183 and 185 eV is identified with the binding energy of Zr 3*d*_5/2 _and 3*d*_3/2 _respectively, shown in Figure 3(c), corresponding to the peaks of Zr^4+ ^ions [27]. The result of Zn 2p core-level XPS spectrum for ZnO (Figure 3(b)) shows that the doublet spectral lines of Zn 2*p *are observed at the binding energy of 1022 eV (Zn 2p_3/2_) and 1045 eV (Zn 2p_1/2_) with a spin-orbit splitting of 23 eV, which coincides with the results for Zn^2+ ^in ZnO [28]. It is important and interesting that the peak in the O 1*s *spectrum (Figure 4(d)) is not totally symmetrical. As reported before, the O 1*s *peak can be fitted by three Gaussion peaks with different binding energy components [29]. The dominant peak located at 530.1 ± 0.2 eV (Oa) is assigned to O^2- ^ions in the ZnO hexagonal wurtzite structure. The medium binding energy component at the peak of 531.2 ± 0.2 eV (Ob) is attributed to lost O^2- ^ions in oxygen deficient regions (oxygen vacancies) within the matrix of ZnO. The highest binding energy component at the peak of 532.4 ± 0.2 eV (Oc) is usually ascribed to nonstoichiometric near-surface oxygen, oxygen atoms in carbonate ions (which are disposed on surfaces of ZnO), surface hydroxylation, adsorbed H_2_O, or adsorbed O_2_. Ob owing to oxygen vacancies, whose area ratio is 22.17%, should be noticed in the above three parts, so we assume that there are a lot of the oxygen vacancies in Zn_0.995_Zr_0.005_O NPs.

**Figure 5:**
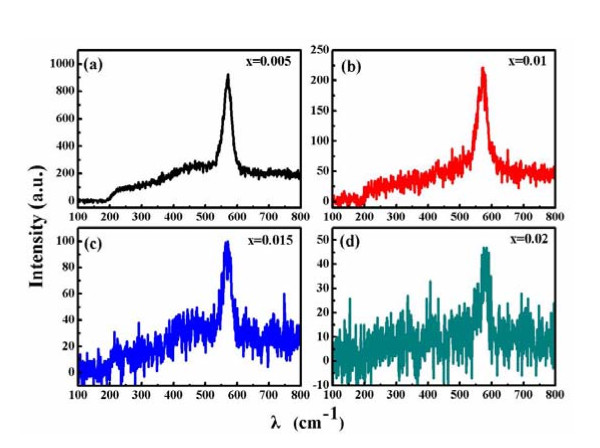
**Raman spectra represented by lines of different colorsof Zn_1-x_Zr_x_O NPs (x = 0.005, 0.01, 0.015, 0.02)**.

The additional information Zn_1-x_Zr_x_O NPs was obtained by Raman spectroscopy. Figure 5 shows the RT Raman spectra of the samples at the range of 100-800 cm^-1^. The sole and obvious peak located at around 574 cm^-1 ^is owing to the A_1 _(LO) phonon mode, which is associated with the defects of oxygen vacancies, Zn-interstitials or their complex [30]. Further, the sole peak from Raman spectra along with the above O 1*s *peak in XPS spectra may be the presence of oxygen vacancies in Zr-doped ZnO lattice.

**Figure 6:**
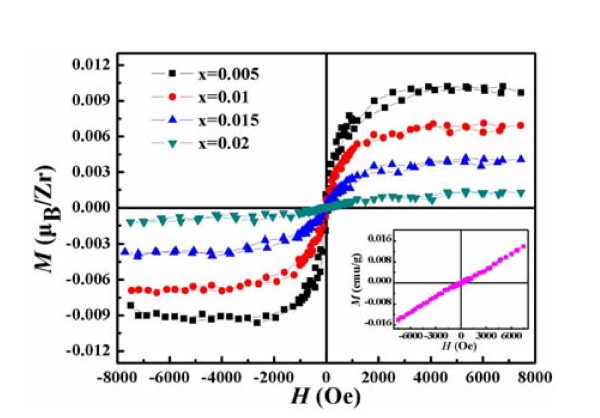
***M-H *curves represented by lines of different colors**. *M-H *curves of Zn_1-x_Zr_x_O NPs (x = 0.005, 0.01, 0.015, 0.02) at RT. The inset is the *M-H *curve of pure ZnO NPs at RT.

The XPS and Raman spectra show there are many oxygen vacancies in samples, oxygen vacancies may cause the RTFM to appear reported before [31,32]. As the result, those motivated us to carry out a comparative study on their magnetic properties. Magnetization curves as a function of applied magnetic field (*M-H*) at RT of samples are revealed in Figure 6, where the contributions of the paramagnetism (PM) signals of the samples were deducted. In the inset of Figure 6, which displays the *M-H *curves of the pure ZnO NPs at RT, the pure ZnO NPs show a PM behavior. Meanwhile it can be seen that the other doping samples exhibit hysteresis curves with the different saturation magnetization (*M_s_*), which indicates that all the doping samples have the clear RTFM. It's sure that the RTFM is induced by doping of Zr. Furthermore, the magnetism of the samples depends strongly on the doping Zr content, and *M_s _*per Zr atom decreases monotonously from 0.0089 *μ_B_*/Zr (Zn_0.995_Zr_0.005_O) to 0.0013 *μ_B_*/Zr (Zn_0.98_Zr_0.02_O) as the increase of the doping content.

**Figure 7:**
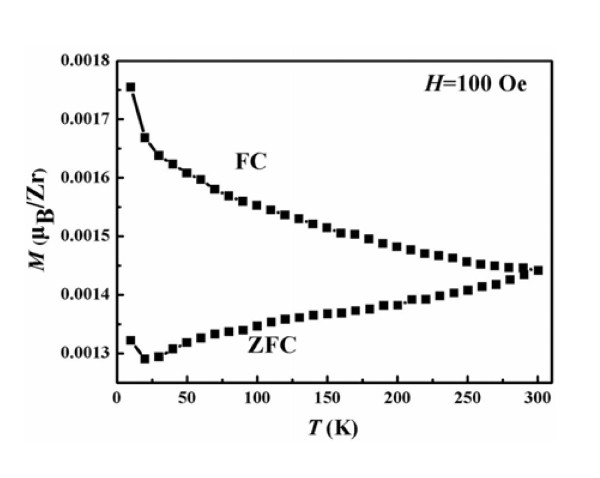
**FC-ZFC curve of Zn_0.995_Zr_0.005_O Nps in the low temperature range of 10-300 K**.

In order to further confirm that there is not any contamination of ferromagnetic cluster formation and the observed FM is the instinct property of Zn_1-x_Zr_x_O NPs, the zero-field-cooled (ZFC) and field-cooled (FC) magnetization curves at the dc field of 100 Oe in the temperature range of 10 to 300 K are measured on these samples, it's given the typical one of Zn_0.995_Zr_0.005_O NPs because of its largest *M_s _*(Figure 7a), which is suggested that there is no blocking temperature. What's more, there is no other FM element (such as Fe, Co) through the XPS with very high precision, because of the above ZFC and FC magnetization curves, the ferromagnetic contamination can be excluded, in other words, the observed RTFM of Zn_1-x_Zr_x_O NPs should be the intrinsic nature. Furthermore, the FC curve exhibits an obvious deviation from the ZFC curve until the temperature above 300 K, indicating that the *T_C _*of the sample is well above 300 K. The result of the ZFC and FC curves suggests the sample has the clear RTFM, which is as the same as the results from VSM.

In other element-doping systems, different mechanisms of FM have been reported. Hou *et al*. reported that the carrier-induced FM (RKKY or double exchange mechanism) might be applied to explain the FM in Cu-doped ZnO films, in which the free carrier concentration is vital to determine whether the material is PM or FM [33]. Meanwhile, Hu *et al*. found that Cr ion substitution is necessary for establishing FM in Cr-doped ZnO films containing V_Zn _[34]. However, Ran *et al*. suggested that defects of Cu-doped ZnO films, such as oxygen vacancies and/or zinc interstitials, might contribute to the RTFM, thus, the observed RTFM was explained in terms of defect-related models [35]. Otherwise, Qi *et al*. concluded that an exchange mechanism associated with oxygen vacancies was responsible for the FM in the Zn_1-x_Er_x_O thin films [23]. At the same time, the RTFM was clearly observed in In-doped ZnO nanowires, which may be associated with oxygen vacancies induced by In doping [36]. In our system, the pure ZnO NPs show the PM behavior, but all of the other doping samples exhibit the clear RTFM, so it's sure that the RTFM is induced by doping of Zr. In the XRD patterns, all the intense peaks from Zn_1-x_Zr_x_O (x = 0.005, 0.01, 0.015, 0.02) could be indexed the same hexagonal wurtzite structure as pure ZnO NPs, the increase in *a *and *c *parameter as a function of Zr concentration is consistent with the substitution of Zn^2+ ^ions (0.74 Å) by Zr^4+ ^ions (0.84 Å) [25,26]. The more Zn^2+ ^were substituted by Zr^4+^, the greater lattice distortion of ZnO would be generated, the more vacancies and/or interstitials should be got. After measured the Raman and XPS, our supposition has been affirmed that there are lots of oxygen vacancies in our samples. As a result, oxygen vacancies should be considered as the origin of FM in our samples, which seems to be similar to the series of Er [23], In [36]-doped ZnO, where the oxygen vacancies also play a crucial role in the RTFM.

## Conclusions

We successfully prepared Zn_1-x_Zr_x_O NPs with the typical pure ZnO hexagonal wurtzite structure by the sol-gel method with post-annealing. All the samples have the clear RTFM, and *M_s _*per Zr atom of samples is sensitive to the content of Zr, and decreases continuously as the increase of the doping Zr content through the magnetic measurement at RT. Combining with the results of Raman and XPS, we suppose that the FM of the Zn_1-x_Zr_x_O NPs is owing to the oxygen vacancies inducing by doping of the nonmagnetic element of Zr.

## Competing interests

The authors declare that they have no competing interests.

## Authors' contributions

JZ prepared the samples, participated in all of the measurements and data analysis, and drafted the manuscript. DG and DX made the conception and design of the manuscript. ZZ2 carried out the XPS measurements and data analysis. JLZ and ZZ1 participated in the XRD measurements and data analysis. GY and ZS participated in the data analysis and the interpretation of the results. All authors have been involved in revising the manuscript, read and approved the final manuscript.

## References

[B1] Dietl T Ohno H Ferromagnetic III-V and II-VI Semiconductors Mrs Bull 2003 28 714 10.1557/mrs2003.211

[B2] Hong NH Sakai J Huong NT Poirot N Ruyter A Role of defects in tuning ferromagnetism in diluted magnetic oxide thin films Phys Rev B 2005 72 045336

[B3] Liu H Zhang X Li LY Wang YX Gao KH Li ZQ Zheng RK Ringer SP Zhang B Zhang XX Role of point defects in room-temperature ferromagnetism of Cr-doped ZnO Appl Phys Lett 2007 91 072511 10.1063/1.2772176

[B4] Dietl T Ferromagnetic semiconductors Semicond Sci Technol 2002 17 377 10.1088/0268-1242/17/4/310

[B5] Chambers SA Ferromagnetism in doped thin-film oxide and nitride semiconductors and dielectrics Surf Sci Rep 2006 61 345 10.1016/j.surfrep.2006.05.001

[B6] Aoki M Yamane H Shimada M Kajiwara T Single crystal growth of manganese gallium nitride using Mn-Ga-Na melt J Alloy Compd 2004 364 280 10.1016/S0925-8388(03)00506-1

[B7] Litvinov VI Dugaev VK Room-temperature ferromagnetism in dielectric GaN(Gd) Appl Phys Lett 2009 94 212506 10.1063/1.3143670

[B8] Cui XY Medvedeva JE Delley B Freeman AJ Newman N Stampfl C Role of Embedded Clustering in Dilute Magnetic Semiconductors: Cr Doped GaN Phys Rev Lett 2005 95 256404 16384484 10.1103/PhysRevLett.95.256404

[B9] Wang Q Sun Q Jena P Kawazoe Y Ferromagnetic to ferrimagnetic crossover in Cr-doped GaN nanohole arrays Phys Rev B 2007 75 075312

[B10] Dietl T Ohno H Matsukura F Cibert J Ferrand D Zener Model Description of Ferromagnetism in Zinc-Blende Magnetic Semiconductors Science 2000 287 1019 10.1126/science.287.5455.1019 10669409

[B11] Yang T Li Y Zhu MY Li YB Huang J Jin HM Hu YM Room-temperature ferromagnetic Mn-doped ZnO nanocrystal synthesized by hydrothermal method under high magnetic field Mater Sci Eng B 2010 170 129 10.1016/j.mseb.2010.03.037

[B12] Chaudhary S Bhatti KP Pandya DK Kashyap SC Nigam AK Effect of indium incorporation in Zn_1-x_Co_x_O thin films J Magn Magn Mater 2009 321 966 10.1016/j.jmmm.2008.03.009

[B13] Tong LN He XM Han HB Hu JL Xia AL Tong Y Effects of H_2 _annealing on ferromagnetism of Ni-doped ZnO powders Solid State Commun 2010 150 1112 10.1016/j.ssc.2010.03.029

[B14] Hong NH Sakai J Brize V Observation of ferromagnetism at room temperature in ZnO thin films J Phys: Condens Matter 2007 19 036219 10.1088/0953-8984/19/3/036219

[B15] Gao DQ Xue DS Xu Y Yan ZJ Zhang ZH Synthesis and magnetic properties of Cu-doped ZnO nanowire arrays Electrochim Acta 2009 54 2392 10.1016/j.electacta.2008.10.051

[B16] Kim CO Kim S Oh HT Choi SH Shon Y Lee S Hwang HN Hwang CC Effect of electrical conduction properties on magnetic behaviors of Cu-doped ZnO thin films Physica B 2010 405 4678 10.1016/j.physb.2010.08.061

[B17] Wang Q Sun Q Jena P Hu Z Note R Kawazoe Y First-principles study of magnetic properties in V-doped ZnO Appl Phys Lett 2007 91 063116 10.1063/1.2768628

[B18] Shi HL Duan YF First-Principles Study of Magnetic Properties of 3*d *Transition Metals Doped in ZnO Nanowires Nanoscale Res Lett 2009 4 480 10.1007/s11671-009-9260-7 20596488 PMC2893854

[B19] Zhuge LJ Wu XM Wu ZF Chen XM Meng YD Effect of defects on room-temperature ferromagnetism of Cr-doped ZnO films Scrirta Mater 2009 60 214 10.1016/j.scriptamat.2008.10.002

[B20] Chawla S Jayanthi K Kotnala RK Room-temperature ferromagnetism in Li-doped *p*-type luminescent ZnO nanorods Phys Rev B 2009 79 125204

[B21] Meyer BK Hofstaetter A Laguta VV Tunneling phenomena of trapped holes in ZnO: Li Physica B 2006 376 682

[B22] Pan H Yi JB Shen L Wu RQ Yang JH Lin JY Feng YP Ding J Van LH Yin JH Room-Temperature Ferromagnetism in Carbon-Doped ZnO Phys Rev Lett 2007 99 127201 17930547 10.1103/PhysRevLett.99.127201

[B23] Qi J Yang YH Zhang L Chi JH Gao DQ Xue DS Room-temperature ferromagnetism in Er-doped ZnO thin films Scrirta Mater 2009 60 289 10.1016/j.scriptamat.2008.10.015

[B24] Paul GK Bandyopadhyay S Sen SK Sen S Structural, optical and electrical studies on sol-gel deposited Zr doped ZnO films Mater Chem Phys 2003 79 71

[B25] Mezdrogina MM Danilevskii EY Kuz'min RV Poletaev NK Trapeznikova IN Chukichev MV Bordovskii GA Marchenko AV Eremenko MV The effect of Fe, Cu, and Si impurities on the formation of emission spectra in bulk ZnO crystals Semiconductors 2010 44 426 10.1134/S1063782610040032

[B26] Fornasiero P Monte RD Rao GR Kaspar J Meriani S Trovaralli A Graziani M Rh-Loaded CeO_2_-ZrO_2 _Solid Solutions as Highly Efficient Oxygen Exchangers: Dependence of the Reduction Behavior and the Oxygen Storage Capacity on the Structural Properties Journal of Catalysis 1995 151 168 10.1006/jcat.1995.1019

[B27] Reddy BM Chowdhury B Reddy EP Fernandez A Characterization of MoO_3_/TiO_2_-ZrO_2 _catalysts by XPS and other techniques J Mol Catal A 2000 162 431 10.1016/S1381-1169(00)00336-8

[B28] Wei XQ Man BY Liu M Xue CS Zhuang HZ Yang C Blue luminescent centers and microstructural evaluation by XPS and Raman in ZnO thin films annealed in vacuum, N_2 _and O_2_ Physica B 2007 388 145 10.1016/j.physb.2006.05.346

[B29] Chen M Wang X Yu YH Pei ZL Bai XD Sun C Huang RF Wen LS X-ray photoelectron spectroscopy and auger electron spectroscopy studies of Al-doped ZnO films Appl Surf Sci 2000 158 134 10.1016/S0169-4332(99)00601-7

[B30] Pradhan AK Zhang K Loutts GB Roy UN Cui Y Burger A Structural and spectroscopic characteristics of ZnO and ZnO:Er^3+ ^nanostructures J Phys: Condens Matter 2004 16 7123 10.1088/0953-8984/16/39/043

[B31] Coey JMD Venkatesan M Fitzgerald CB Donor impurity band exchange in dilute ferromagnetic oxides Nat Mater 2005 4 173 10.1038/nmat1310 15654343

[B32] Herng TS Qi DC Berlijn T Yi JB Yang KS Dai Y Feng YP Santoso I Sanchez-Hanke C Gao XY Wee ATS Ku W Ding J Rusydi A Room-Temperature Ferromagnetism of Cu-Doped ZnO Films Probed by Soft X-Ray Magnetic Circular Dichroism Phys Rev Lett 2010 105 207201 21231259 10.1103/PhysRevLett.105.207201

[B33] Hou DL Ye XJ Zhao XY Meng HJ Zhou HJ Li XL Zhen CM Room-temperature ferromagnetism in n-type Cu-doped ZnO thin films J Appl Phys 2007 102 033905 10.1063/1.2764203

[B34] Hu YM Hsu CW Wang CY Lee SS Wang SJ Han TC Chou WY Room-temperature ferromagnetism in co-sputtered Zn_1-x_Cr_x_O films with low Cr content Scrirta Mater 2009 61 1028 10.1016/j.scriptamat.2009.08.018

[B35] Ran FY Tanemura M Hayashi Y Hihara T Effect of substrate temperature on the room-temperature ferromagnetism of Cu-doped ZnO films J Cryst Growth 2009 311 4270 10.1016/j.jcrysgro.2009.07.008

[B36] Liu KW Sakurai M Aono M Indium-doped ZnO nanowires: Optical properties and room-temperature ferromagnetism J Appl Phys 2010 108 043516 10.1063/1.3464229

